# Acute Oral Toxicity and Brine Shrimp Lethality of *Elaeis guineensis* Jacq., (Oil Palm Leaf) Methanol Extract

**DOI:** 10.3390/molecules15118111

**Published:** 2010-11-10

**Authors:** Abdul Rani Muhamad Syahmi, Soundararajan Vijayaratna, Sreenivasan Sasidharan, Lachimanan Yoga Latha, Yuet Ping Kwan, Yee Ling Lau, Lai Ngit Shin, Yeng Chen

**Affiliations:** 1 School of Biological Sciences, Universiti Sains Malaysia, 11800, Pulau Pinang, Malaysia; 2 Institute for Research in Molecular Medicine (INFORMM), Universiti Sains Malaysia, 11800, Pulau Pinang, Malaysia; E-Mails: latha_usm@yahoo.com (L.Y.L.); chenyeng@usm.my (Y.C.); 3 Department of Parasitology, Faculty of Medicine, University of Malaya, 50603 Kuala Lumpur, Malaysia; E-Mail: lauyeeling@um.edu.my (Y.L.L.)

**Keywords:** *Elaeis guineensis*, methanol extract, acute oral toxicity, brine shrimp lethality assay

## Abstract

*Elaeis guineensis* (Arecaceae) is widely used in West African traditional medicine for treating various ailments. An evaluation on the toxicity of extracts of this plant is crucial to support the therapeutic claims. The acute oral toxicity and brine shrimp lethality of a methanolic extract of this plant was tested. Oral administration of crude extract at the highest dose of 5,000 mg/kg resulted in no mortalities or evidence of adverse effects, implying that *E. guineensis* is nontoxic. Normal behavioral pattern, clinical signs and histology of vital organs confirm this evidence. The *E. guineensis* extracts screened for toxicity against brine shrimp had 50% lethal concentration (LC_50_) values of more than 1.0 mg/mL (9.00 and 3.87 mg/mL, at 6 and 24 h, respectively), confirming that the extract was not toxic. Maximum mortalities occurred at 100 mg/mL concentration while the least mortalities happened to be at 0.195 mg/mL concentration. The results of both tests confirm that *E. guineensis* is nontoxic and hence safe for commercial utilization.

## 1. Introduction

Traditional and alternative medicine is extensively practiced in the prevention, diagnosis, and treatment of various illnesses. It has regained public attention over the past 20 years as this type of medicine is easily accessible in some regions. A postulation denoted that by 2010, at least two-thirds of Americans will opt for alternative therapies [1].

*Elaeis guineensis* or oil palm is among the plants that are widely used by the traditional natives of West Africa. All parts of this plant are useful. The wood is used as frames for buildings, and the sap is fermented into palm wine. The oil from the fruit mesocarp and the seeds are used for cooking, and making soaps, creams, and other cosmetics. The fresh sap is used as a laxative, and partially fermented palm wine is administered to nursing mothers to improve lactation. The fruit husk is used in the preparation of soaps used to treat skin infections. A root decoction is used to treat headaches in Nigeria. The pulverized roots are added to drinks as a cure for gonorrhea, menorrhagia, and bronchitis [2]. The leaf extract and juice from young petioles are applied to fresh wounds. The fruit mesocarp oil and palm kernel oil are administered as a poison antidote and used externally with several other herbs as a lotion to treat skin diseases. Palm kernel oil is applied to convulsive children to regulate their body temperature. Oil palm is a folk remedy for cancer, headaches, and rheumatism, and is considered an aphrodisiac, a diuretic, and a liniment [2]. In Liberia, split leaf stems of the oil palm are woven as splints [3]. Investigations on functional plants provide evidence on the presence of substances that are potential for human health benefits. However, there should be a vital requirement to determine the toxic effects of some of the substances contained in the plants [4].

Toxicity is an expression of being poisonous, indicating the state of adverse effects led by the interaction between toxicants and cells. This interaction may vary depending on the chemical properties of the toxicants and the cell membrane, as it may occur on the cell surface, within the cell body, or in the tissues beneath as well as at the extracellular matrix. The toxic effects may take place prior to the binding of the toxicants to the vital organs such as liver and kidneys. Hence, evaluation of toxic properties of a substance is crucial when considering for public health protection because exposure to chemicals can be hazardous and results to adverse effects on human being. In practice, the evaluation typically includes acute, sub-chronic, chronic, carcinogenic and reproductive effects [5]. 

In the present study of the methanol extracts of *E. guineensis* leaf, both the acute oral toxicity test in animal models [6] and the brine shrimp lethality test [7] were applied to determine its toxic properties. The acute oral toxicity testing was carried out for both sexes of animals under the Organization for Economic Cooperation and Development (OECD) guidelines [8].

## 2. Results

### 2.1. Lethality and Behavioral Analysis

The lethality and toxicity effect of the methanol extracts of *Elaeis guineensis* on the appearance and behavioral pattern mice are shown in Table 1 and Table 2, respectively. There were no deaths among the animals during the observation period and no significant changes in general appearance or behavioral pattern were noted either. Moreover, all the organs either of the control or the test groups were in good shape and condition.

### 2.2. Organs and Body Weight Statistical Analysis

The body weight as well as the weights of the vital organs of the animals were calculated and recorded in Table 3. There were no significant differences in the changes of each weight.

### 2.3. Histopathology Analysis of Heart, Kidneys, Liver, Lung, Spleen, and Ribcage

**Figure 1:**
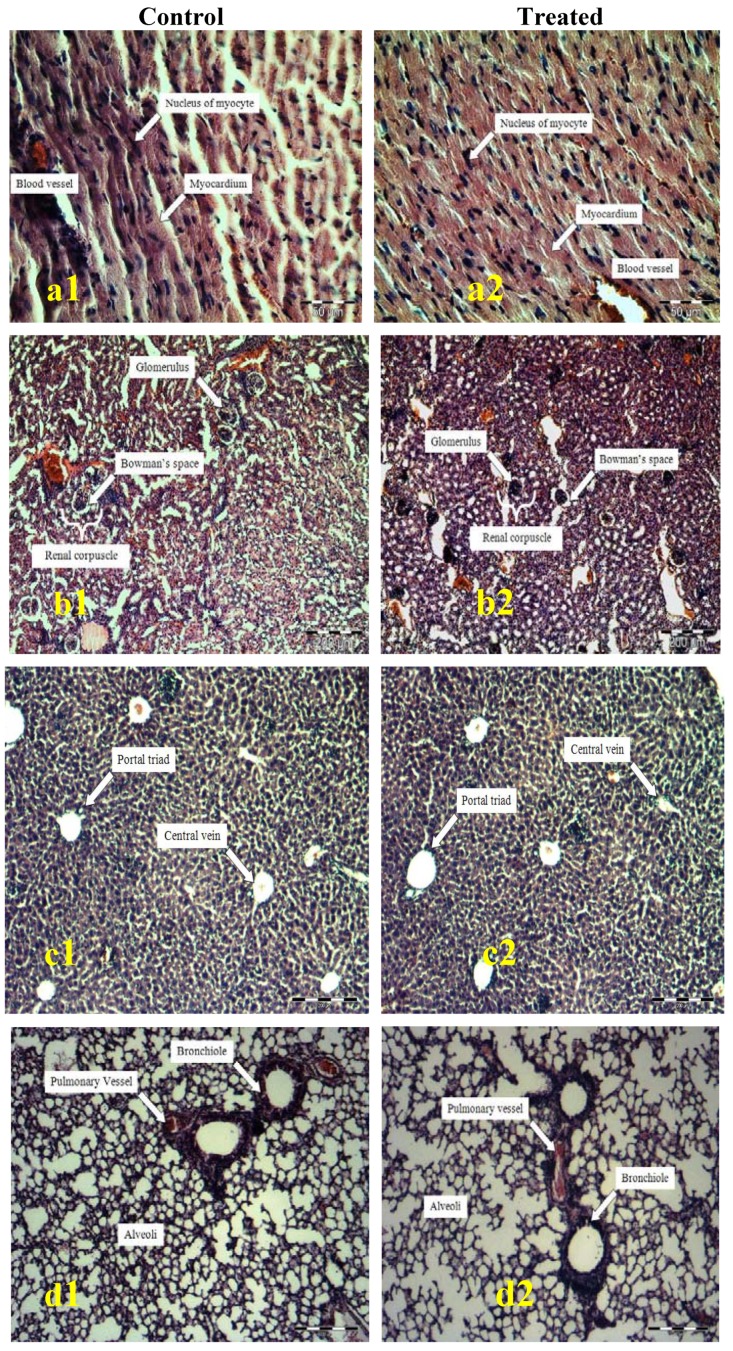

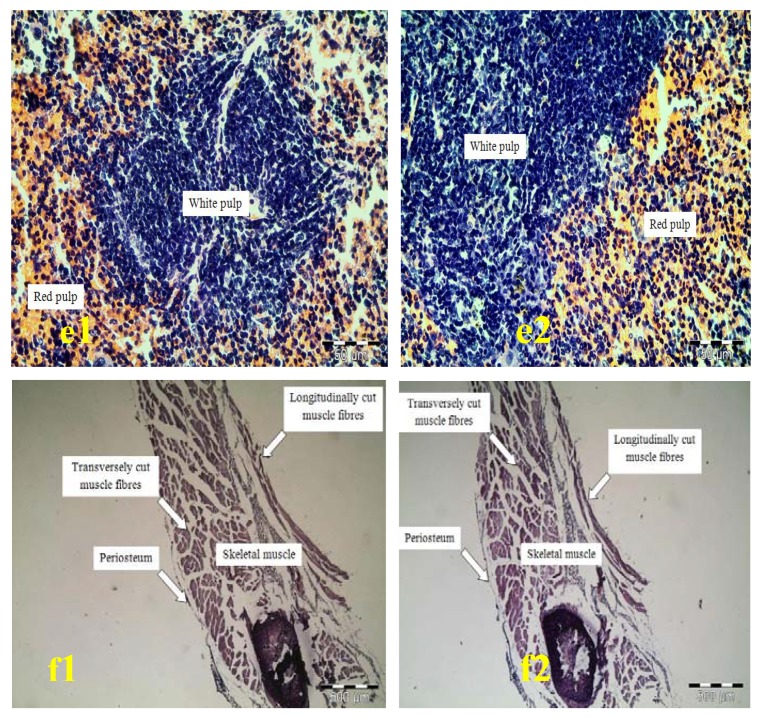
Histological examination of heart (a), kidneys (b), liver (c), lung (d), spleen (e) and ribcage (f).

The microscopic structures of the organs depicted through Figure 1 shows unnoticeable differences between the control and test group. There were also no cell degradation or any unfavorable effects observed when viewed under the light microscope using multiple magnification power.

### 2.4. Brine Shrimp Lethality Test

**Figure 2:**
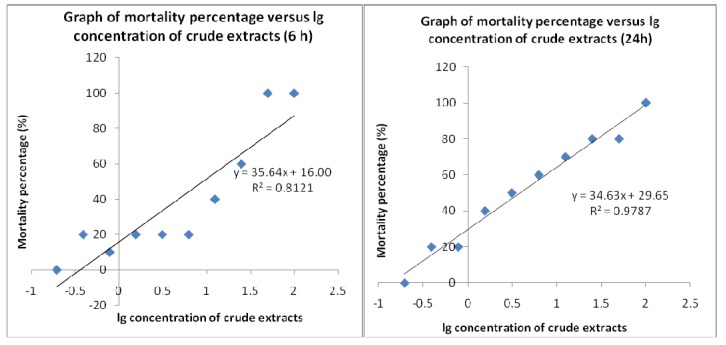
Brine shrimp lethality of *Elaeis guineensis* crude extracts at 6 h and 24 h.

**Figure 3:**
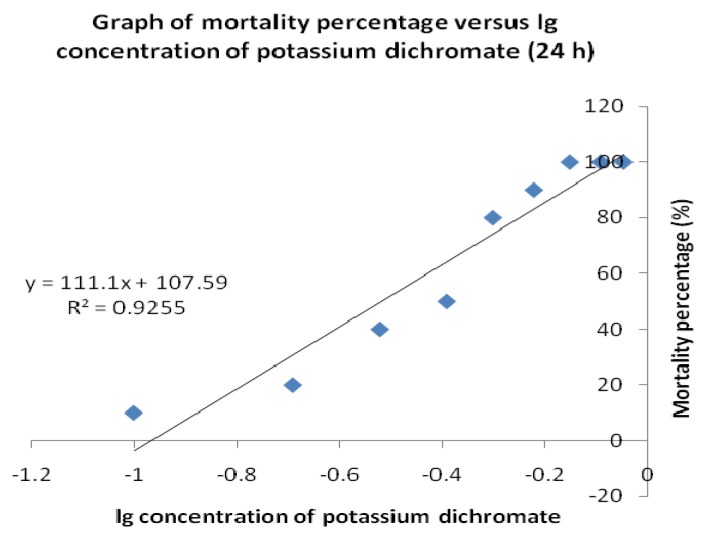
Brine shrimp lethality of potassium dichromate as a positive control at 24 h.

Brine shrimp lethality results of the methanolic crude extracts of *E. guineensis* are shown in Figure 2a, 2b and Figure 3 and the LC_50_ values calculated are recorded in Table 4. The methanolic crude extracts show positive results, indicating that the samples are biologically active. Crude extracts resulting in LC_50_ values of less than 1 mg/mL are considered as significantly active which suggests that the *E. guineensis* crude extracts, with LC_50_ values of 9.00 and 3.87 mg/mL at 6 and 24 hour, respectively, have a very low toxicity. Plotting of mortality percentage *versus* log of concentration for all tests (Figure 2a, 2b and Figure 3) demonstrates an approximate linear correlation. Furthermore, there is a direct proportional relation between the concentration of the extracts and the degree of lethality. This is shown by the fact the maximum mortalities occurred at a concentration of 100 mg/mL whilst a concentration of 0.195 mg/mL only caused very minor mortalities.

Brine shrimp lethality results of the potassium dichromate are shown in Figure 3 and the LC_50_ values calculated are recorded in Table 4. Potassium dichromate served as the positive control for this brine shrimp lethality assay. The LC_50_ values for the positive control at 24 hours were 0.30 mg/mL, has shown that it exhibits toxic expressions (LC_50_ was less than 1.0 mg/mL) against the brine shrimp. 

Nevertheless, as toxic compound expressions can be insignificant *in vivo*, further investigation of this compound by using *in vitro* method should be pursued. Based on the collective results, the leaf of *E. guineensis* exhibits no acute toxic effects against the animals, and hence signifying that this plant is also not toxic to humans.

## 3. Discussion

The acute oral toxicity of *E. guineensis* leaf methanol extracts was determined in the present study. The evaluations of the *in vivo* toxicity were done both qualitatively and quantitatively by performing a histopathology study as well as determining the LC_50_ value using the brine shrimp lethality test. As use of medicinal plants increases, experimental screening of the toxicity of these plants is crucial to assure the safety and effectiveness of those natural sources. In general, *in vivo* methods are likely to provide an early hint of toxicity of a compound since applying *in vitro* cytotoxicity methods could result in limited information. By applying *in vivo* assays, toxic expressions such as pain, distress, allergic reactions, physical changes and behavioural alterations in the tested animals can be detected. However, acute toxicity studies do not detect effects on vital functions like the cardiovascular, central nervous, and respiratory systems which are not usually assessed during the study and these should be evaluated prior to human exposure.

### 3.1. Acute Oral Toxicity Study on Animal Models

Investigation of acute toxicity is the first step in the toxicological analysis of any medicinal plant. Oral acute toxicity testing in mice could be used to evaluate natural remedies for different pharmacological activities, taking into account the basic premise that pharmacology is simply toxicology at a lower dose [9]. A toxic substance might elicit interesting pharmacological effects at a lower non-toxic dose. Toxicity results from animals will be crucial in definitively judging the safety of *E. guineensis* extracts if they are found to have sufficient potential for development into pharmacological products [10]. 

In this study of acute oral toxicity, 40 Swiss albino mice from both sexes were employed to observe the toxicity effects of methanol crude extract of *E. guineensis* leaves. The route of administration depends on the dosage form in which the compound is available. Based on historical research, the oral route administration is the most convenient and commonly used one when studying acute toxicity. The absorption might be slow, but this method costs less and is painless to the animals. Since the crude extract is administered orally, the animals should be fasted before taking the dose because food and other chemicals in the digestive tracts may affect the reaction(s) of the compound. 

Although there is a problem regarding extrapolating animals data to humans, a study has shown that mice give better prediction for human acute lethal dose compared to rats [11]. All the procedures were performed based on the appropriate OECD guideline [8,12]. Because it is generally known that *E. guineensis* leaf is edible to animals, hence it is preliminarily assumed to be not toxic. Therefore in this limit dose study, a very high doses level of 5,000 mg/kg of crude extracts were administered orally to the tested mice (OECD) [8,12]. 

From the current testing, no mortalities were reported as well as no adverse effects were observed on the tested mice throughout the 14 day test period. All of the mice gained weight and displayed no significant changes in behavior. The physical appearance features such as skin, fur and eyes were found to be normal and whilst as the body weight of the mice showed as increase, this indicates that the administration of the crude extract does not affect the growth of the animals. Thus, this test reckoned that *E. guineensis* does not cause acute toxicity effects and an LD_50_ value greater than 5,000 mg/kg. In principle, the limit test method is not intended for determining a precise LD_50_ value, but it serves as a suggestion for classifying the crude extract based on the expectation at which dose level the animals are expected to survive [13]. Therefore, according to the chemical labeling and classification of a cute systemic toxicity recommended by OECD, the crude extract of *E. guineensis* was assigned class 5 status (LD_50_ > 5000 mg/kg) which was the lowest toxicity class.

Based on the histopathology analysis, all of the tissues of organs presented good structures with no cellular lesions being observed. There were no significant differences when comparing both the slides of the organs from tested animals and the controls, which suggesting that the crude extracts did not interact with the target cells or change the biological systems of the animals. The present results suggest the possibility of this extract as a potential source for the development of pharmacological agent to treat various types of ailment.

### 3.2. Brine Shrimp Lethality Test

Brine shrimp bioassay is considered as a rapid preliminary screening for the presence of biochemical activity and was used to determine the crude extract’s toxicity. This test is based on the potential of *E. guineensis* methanol extract to become lethal to *A. salina* nauplii due to its toxic expression. According to Meyer *et al*. [7], extracts derived from natural products which have LC_50_ ≤ 1.0 mg/mL are known to possess toxic effects. In this study, the plotted graphs show that the LC_50_ value of the crude extract is 9.00 and 3.87 mg/mL for 6 and 24 h, respectively. Thus, these results prove that the methanol extracts of *E. guineensis* are not toxic and show that the *E. guineensis* leaf extract may be further explored for the development of natural product-based pharmaceutical products.

## 4. Experimental 

### 4.1. Plant Material

Fresh samples of *E. guineensis* leaves was obtained from Kampung Lekir, Sitiawan, Perak, Malaysia in August 2009 and authenticated by Mr. Shunmugam A/C Vellosamy from the Herbarium Unit, School of Biological Science, Universiti Sains Malaysia. The dried parts of the plant including leaves, fruits and flowers were deposited as voucher specimens (with herbarium number 11036) at the Herbarium Unit, School of Biological Science. 

### 4.2. Preparation of the Crude Extracts

The midribs of the *E. guineensis* leaves were removed before cutting the leaflets into pieces. The sample was then washed thoroughly and rinsed with tap water and dried in oven at 60 °C for two to four days. The leave sample was sequentially extracted with methanol by adding approximately 100 g of the dried sample (in fine powder form) to 400 mL methanol. The extraction was carried out at room temperature by soakinbg for four days with intermittent stirring during the first day. The extracts were filtered and the process of extraction was repeated again for a second time by adding another 400 mL of solvent to the sample residue. The filtrate from each extraction was combined and concentrated under vacuum by rotary evaporator until a dark green methanol extract was produced. The extracts were freeze dried and kept at 4 °C until use.

### 4.3. Acute Oral Toxicity Study 

#### 4.3.1. Target Organisms-Mice

The experiment was conducted on 40 healthy Swiss albino mice (males and females) weighing 25 to 35 g and aged 8 to 10 weeks, acquired from the Animal House, Universiti Sains Malaysia. Those mice were distributed into four groups, *i.e.*, two treated groups and two control groups of opposite sex. The experimental procedures relating to the animals were authorized Universiti Sains Malaysia Ethical committee before starting the study and were conducted under the internationally accepted principles for laboratory animal use and care (EEC Directive of 1986; 86/609/EEC).

#### 4.3.2. Toxicity Test

The mice used in the experiment were selected at random and marked on the tails for individual identification. Ten mice of the same sex were kept in a matte plastic cage, with dimensions of 17 × 27 × 14 cm. All of the cages were located in a room at temperature approximately 23 °C with constant humidity. The room is regulated with cycles of 12 h of light and 12 h of darkness. The mice were acclimated to the laboratory environment for a week earlier before starting the experiment. Drinking water and food were provided *ad libitum* through the experiment, except for the short fasting period where the drinking water was still in free access but no food supply was provided 12 h prior to treatment. The acute oral toxicity of *E. guineensis* methanol crude extracts was evaluated in mice according to the procedures outlined by the Organization for Economic Co-operation and Development (OECD) [8,12]. A single high dose of 5,000 mg/kg of crude extracts was administered to both ten male mice and ten female mice through the oral route. The extracts were suspended in a vehicle (Tween-20 in distilled water). Following the fasting period, body weight of the mice was determined and the dose was calculated in reference to the body weight as the volume of the extracts solution given to the mice is 10 mL/kg. Another ten male mice and ten female mice were allotted distilled water and were regarded as the control groups. Food was provided to the mice approximately an hour after treatment. The mice were observed in detail for any indications of toxicity effect within the first six hours after the treatment period, and daily further for a period of 14 days. Surviving animals were weighed and visual observations for mortality, behavioral pattern, changes in physical appearance, injury, pain and signs of illness were conducted daily during the period.

### 4.4. Histological Analysis

#### 4.4.1. Organs and Body Weight Statistical Analysis

Finishing the 14 day period, all the mice were sacrificed. Vital organs such as heart, kidneys, liver, lung and spleen, and also a fragment of the rib cage were isolated and examined for any lesions. All of the individual organs were weighed and their features were compared between both treated and control groups. Statistical analysis to assess the significant difference between both groups was conducted by running a T test using Microsoft Excel spreadsheet application. The level of significance used in this analysis is 5%.

#### 4.4.2. Histopathology of Heart, Kidneys, Liver, Lung, Spleen, and Ribcage

All the vital organs and the rib cages isolated from each individual were fixed in 10% buffered formalin, routinely processed and embedded in paraffin wax. Paraffin sections (5 µm) were cut on glass slides and stained with haematoxylin and eosin. The slides were examined under a light microscope and the magnified images of the tissues structure were captured for further study [14].

### 4.5. Brine Shrimp Lethality Test

#### 4.5.1. Hatching Shrimp

Brine shrimp eggs, *Artemia salina* were hatched in a vessel containing sterile artificial seawater prepared by dissolving 38 g table salt in 1 L distilled water. The vessel was kept under an inflorescent bulb and facilitated with good aeration for 48 h at room temperature. After hatching, nauplii released from the egg shells were collected at the bright side of the vessel (near the light source) by using micropipette. The larvae were isolated from the eggs by aliquoting them in small beaker containing the seawater.

#### 4.5.2. Brine Shrimp Test

The bioactivity of the extracts was monitored by the brine shrimp lethality test [7] to predict the presence of cytotoxic activity in the compound. The extracts was dissolved in methanol and diluted with artificial seawater. The assay system was conducted by preparing 10 bijoux bottles filled with 2 mL of seawater each and a two-fold dilution was set up to yield a series of concentrations from 100 to 0.195 mg/mL. Potassium dichromate was dissolved in artificial seawater and functioned as a positive control with concentrations ranging from 0.1 to 0.9 mg/mL. An aliquot (0.1 mL) containing about 10 to 15 nauplii was introduced to each bottle and the setup was allowed to continue for 24 h. the bottles were observed, and the dead larvae from each bottles were counted after 6 and 24 h. Based on the percentage of the mortality, the concentration that led 50% lethality (LC_50_) to the nauplii was determined by using the graph of mean percentage mortality *versus* the log of concentration [15].

#### 4.5.3. Statistical Analysis

The mean results of mortality percentage of the brine shrimp *versus* the log of concentrations were plotted using the Microsoft Excel spreadsheet application, which also formulated the regression equations. These equations were later used to calculate LC_50_ values for the samples tested with consideration of value greater than 1.0 mg/mL, suggesting that the extract is nontoxic.

## 5. Conclusions 

Our results suggest that *E. guineensis* methanol extract does not cause any apparent *in vivo* toxicity in an animal model. The results of the current study concur with the use of this plant by traditional healers, especially in Africa. A Word Health Organization survey indicated that about 70-80% of the world’s population rely on non-conventional medicine, mainly from herbal sources, in their primary healthcare, hence *E. guineensis* may be used as a medicinal agent in known dosages, especially in rural communities where conventional drugs are unaffordable because of the high cost. Studies of this type are needed before a phytotherapeutic agent can be generally recommended for pharmaceutical use.

## Figures and Tables

**Table 1:** Potential toxic effects of the crude extracts of *Elaeis guineensis* in mice.

Male		Female	
Control^a^	Crude extract^b^	Control	Crude extract
0/10^c^	0/10	0/10	0/10

^a^ Control group (treatment without crude extract); ^b^ Test group (treatment with 5000 mg/kg crude extract); ^c^ Number of dead mice/number of mice used.

**Table 2:** General appearance and behavioral observations for control and treated groups.

Observations	Control group		Test group	
	6 h	14 day	6 h	14 day
Skin and fur	Normal	Normal	Normal	Normal
Eyes	Normal	Normal	Normal	Normal
Mucous membrane	Normal	Normal	Normal	Normal
Behavioral patterns	Normal	Normal	Normal	Normal
Salivation	Normal	Normal	Normal	Normal
Lethargy	Normal	Normal	Normal	Normal
Sleep	Normal	Normal	Normal	Normal
Diarrhea	Normal	Normal	Normal	Normal
Coma	N.O.^a^	N.O.	N.O.	N.O.
Tremors	N.O.	N.O.	N.O.	N.O.

^a^ Not Observed.

**Table 3:** Effect of *Elaeis guineensis* crude extract on organ-to-body weight index (%) in mice.

	Male		Female	
Organ	Control	Crude extract	Control	Crude extract
Heart	0.54 ± 0.06	0.63 ± 0.07	0.58 ± 0.05	0.61 ± 0.06
Kidneys	1.63 ± 0.04	1.68 ± 0.05	1.61 ± 0.06	1.40 ± 0.08
Liver	6.33 ± 0.19	6.43 ± 0.14	6.35 ± 0.17	5.94 ± 0.17
Lung	1.21 ± 0.04	1.20 ± 0.05	1.12 ± 0.06	0.90 ± 0.07
Spleen	0.47 ± 0.06	0.51 ± 0.05	0.48 ± 0.05	0.48 ± 0.08
Body Weight (g)	31.22 ± 0.89	31.90 ± 0.70	31.21 ± 0.76	28.08 ± 0.66

Organ body index = (organ weight × 100)/body weight; acrude extract of *Elaeis guineensis* was administered to mice at a dose of 5000 mg/kg; values are mean ± SD (n = 10) at 5% level of significance.

**Table 4:** Brine shrimp toxicity expressed as LC_50_ value.

Sample	LC_50_ (mg/mL)
*Elaeis guineensis* (6 h)	9.00
*Elaeis guineensis* (24 h)	3.87
Potassium dichromate (24 h)	0.30
